# Use and Comparison of Machine Learning Techniques to Discern the Protein Patterns of Autoantibodies Present in Women with and without Breast Pathology

**DOI:** 10.1021/acs.jproteome.4c00759

**Published:** 2024-12-19

**Authors:** José-Luis Llaguno-Roque, Rocio-Erandi Barrientos-Martínez, Héctor-Gabriel Acosta-Mesa, Antonia Barranca-Enríquez, Efrén Mezura-Montes, Tania Romo-González

**Affiliations:** 1 Laboratorio de Biología y Salud Integral, Instituto de Investigaciones Biológicas − Universidad Veracruzana, Dr. Luis Castelazo Ayala S/N, Industrial Animas. C.P., Xalapa, Veracruz 91190, México; 2 Instituto de Investigaciones en Inteligencia Artificial − Universidad Veracruzana, Campus Sur, Calle Paseo Lote II, Sección Segunda N° 112, Nuevo Xalapa, C.P., Xalapa, Veracruz 91097, México; 3 Centro d Estudios y Servicios en Salud − Universidad Veracruzana, Carmen Serdan Esq. F. J. Mina 147 Col. Flores Magón C.P., Veracruz, Veracruz 91700, México

**Keywords:** artificial intelligence, neuroevolution, time series, western blot, autoantibodies

## Abstract

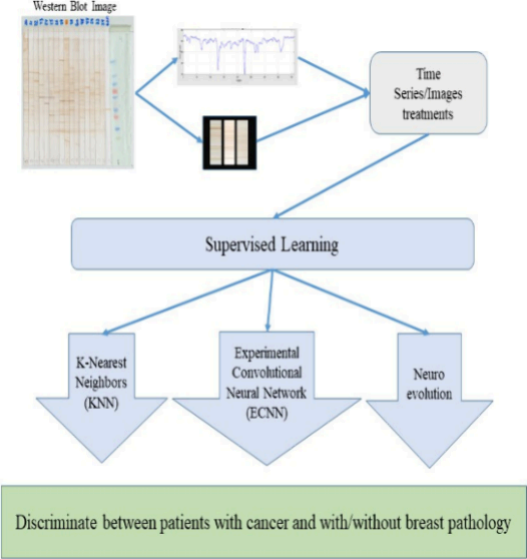

Breast cancer (BC) has become a global health problem, ranking first in incidence and fifth in mortality in women around the world. Although there are some diagnostic methods for the disease, these are not sufficiently effective and are invasive. In this work, we discriminated between patients without breast pathology (BP), with benign BP, and with BC based on the band patterns obtained from Western blot strip images of the autoantibody response to antigens of the T47D tumor line using and comparing supervised machine learning techniques to have a sensitive and accurate method. When comparing the aforementioned machine learning techniques, it was found that by obtaining a convolutional neural network architecture from a neuroevolution algorithm, it is possible to automatically discriminate with a classification accuracy of 90.67% between patients with cancer and with/without BP. In the case of discrimination between patients with cancer and without BP, a classification accuracy of 96.67% was obtained with the K-NN algorithm and 95.13% with the convolutional neural network obtained using a neuroevolution algorithm, although these results are not statistically significant. It is concluded that the convolutional neural network obtained by neuroevolution is the method with the best performance with respect to those evaluated in this work.

## Introduction

Breast cancer is a disease that has become a global health problem. In terms of incidence, it ranks first, estimated in 2022 at 2.3 million new cases, that is, 23.8% of all cancer cases worldwide. In addition, it ranks first in cancer mortality worldwide with 667,000 deaths. In Mexico, breast cancer also represents a challenge in the health sector, since the incidence of this disease in 2022 was 31,043 new cases; registering 7,839 new cases for the age range of 20 to 44 years, 14,846 incidents for ages between 40 and 59 years, and 11,430 new cases for those over 60 years of age.^1^ The first two age ranges (20–59 years) are the productive stages of women, which represents a loss in the work aspect, social, family and economic environment.^2^ Furthermore, breast cancer in Mexico occurs in women at 52.3 years of age, a decade earlier than in developed countries. Regarding mortality, in Mexico in 2022 there were 8,195 deaths due to breast cancer. In the range of 20 to 44 years there were 1,162 deaths, for ages between 40 and 59 years there were 3,555 and for those over 60 years of age, 4,061 women succumbed to this disease. As can be seen in the figures, the older you get, the mortality rate rises.^1^

Breast cancer is a pathology characterized by the accelerated and uncontrolled dissemination, proliferation and reproduction of mammary epithelial cells, forming tumors, which depending on their characteristics can be malignant or benign. Malignant tumors can be divided into two types according to the degree of invasion: A) In situ, located in the ducts or lobules of the breast, without invasion of the stroma. This type of cancer includes ductal carcinoma and lobular carcinoma, with the former being the most common, representing between 70% and 80% percent of cases. B) Infiltrating or invasive, this type of tumor penetrates the fat of the breast duct, also reaching the blood and lymphatic vessels. Invasive ductal and invasive lobular carcinoma are found in this type of tumors.^2,3^

Cancerous tumors, and therefore breast tumors, develop because they can evade the immune system. That is, cancer cells evade the antitumor response of the immune system, which cannot determine in advance whether the cell is its own or foreign, allowing the tumor to reorganize the surrounding tissue, create its own microenvironment and change the extracellular matrix, blood vessels, and various cells, such as supporting cells, stromal cells, immune cells, and endothelial cells, which together contribute to tumor progression.^4^

In Mexico, the commonly used breast cancer detection methods are clinical examination of the breasts, ultrasound, mammography, and biopsy is used as a confirmatory test for the disease. On the other hand, there are detection methods that are available, but they are not yet fully accepted such as thermography.^5^ The disadvantages of these methods mentioned previously are that they are invasive, painful, expensive and subjective (they require the analysis of an expert) and are generally performed when the disease is in an advanced stage.^6^ Therefore, detection of breast cancer should occur in the early stages of development, so that patients receive a timely diagnosis and a better treatment and prognosis. Therefore, the reaction of autoantibodies to tumor autoantigens presents an alternative in the early detection of breast cancer.^7^ Breast cancer tumor cells produce tumor-associated antigens (TAA). These TAAs may have abnormal structures that the immune system no longer recognizes as their own, causing the stimulation of B and T lymphocytes to produce specific autoantibodies directed against these TAAs. Autoantibodies are amplified (cloned) by humoral immunity, causing patients to have more autoantibodies than TAAs in the peripheral blood.^8^

The characteristics that autoantibodies have lead them to become candidates for biomarkers for the early detection of breast cancer. These characteristics are 1) Autoantibodies have a half-life of 7–30 days, being more stable than other serum proteins. 2) Autoantibodies can be detected before the tumor produces clinical symptoms. 3) The patient’s breast density does not affect the detection of autoantibodies in peripheral blood. Tumor-associated autoantibodies such as p53, MUC1, and HER2 could be used as protein biomarkers in breast cancer tumors.^9,10^ The production of autoantibodies against tumor-associated autoantigens, as a response by the immune system, occurs at an early stage of tumorigenesis, causing the autoantibodies to be detected before the clinical symptoms of breast cancer manifest, thereby decreasing the mortality of patients with this condition.^11^

In the medical field, artificial intelligence has contributed to the process of preventing and diagnosing diseases such as breast cancer, using techniques such as machine learning and computer vision. Which are designed, for example, to identify patterns in images (magnetic resonance imaging (MRI), thermographic, mammography, etc.), as well as the classification of women with/without breast lesions.^5,12,13^ For example, in the work of Zhu et al., (2022)^14^ they use convolutional neural networks to diagnose breast cancer, classifying time series acquired from taking temperature and humidity from sensors in the breast. In Gardezi et al., (2018),^15^ they obtain time series of an area of interest from mammographic images, with the objective of characterizing normal and abnormal tissue through classification algorithms and using the dynamic time warping (DTW) technique as a similarity metric. between the time series. In José Escorcia-Gutierrez et al., (2022) they use convolutional neural networks in mammographic images to detect breast lesions. In the work of Abunasser et al., (2023)^16^ used images of breast cancer tissue to detect and classify different types of breast pathologies by evaluating the performance of various convolutional neural network architectures.

Sánchez-Silva et al. (2018),^17^ proposed a semiautomatic tool to diagnose breast cancer, based on the classification of time series obtained from Western Blot images of the autoantibody reaction to antigens of a tumor line. To classify the time series, the K-Nearest Neighbor (KNN) algorithm was used, using Euclidean, Mahalanobis and correlation similarity distances, achieving a classification accuracy of 65.40% with three classes (healthy, benign breast pathology and breast cancer), and a classification accuracy of 86.06% with two classes (healthy and breast cancer). The classification percentages achieved are similar to those of the reference expert.^18^ However, the method is considered semiautomatic since to obtain the time series, an area is subjectively selected in each band, which causes variation in the lengths of the time series and needs to be standardized. To improve these results, it was proposed to analyze the image of each Western Blot strip of each patient, which contain the bands of the antibody reaction to antigens (tumor line T47D—ductal carcinoma), using a convolutional neural network whose architecture was obtained experimentally, achieving a classification accuracy of 66.89% for three classes of patients (Without breast pathology, with benign breast pathology, with cancer), and for two classes (Without breast pathology, with cancer) an accuracy of 86.00%.^19^ These results, although improved compared to those reported in Sánchez-Silva et al. (2018),^17^ did not show a statistically significant difference. Therefore, to improve these results, the neuroevolution algorithm (DeepGA) was used to obtain a convolutional neural network (CNN) architecture,^20^ which allowed a satisfactory execution with an automatic classification accuracy of 90.67% for only three classes (Without breast pathology, With benign breast pathology, With cancer).^21^ Although the result obtained was quite high with respect to the literature,^17,19^ and was obtained automatically, given the importance of having highly sensitive and accurate methods for the diagnosis of breast cancer, it is convenient to analyze other tools and compare the results with those obtained previously.

Based on the above, this paper proposes to use supervised learning (part of machine learning) using the K-nearest neighbors algorithm and Dynamic Time Warping (DTW) as a similarity measure for the analysis of one-dimensional Western Blot images containing the expression of bands of the autoantibody reaction to antigens of the T47D tumor line (ductal carcinoma), and to compare their performance with the architectures of convolutional neural networks obtained experimentally and through neuroevolution described in the literature,^19,21^ in order to identify the ideal method to classify patients without breast pathology, with benign breast pathology and breast cancer for the early detection of breast cancer.

## Materials and Methods

### Participants

Mexican women between 16 and 79 years of age were invited to participate in this study through an open call at the Institute of Biomedical Research (UNAM, Mexico) or were invited to participate at their first gynecologic-oncologic appointment (before any previous oncologic treatment) at the General Hospital of Mexico “Dr. Eduardo Liceaga”. The protocol for obtaining blood samples from participants was reviewed and approved by the Research Ethics Committee of the General Hospital of Mexico “Dr. Eduardo Liceaga” (DI/12/111/03/064), and complies with the Code of Ethics of the World Medical Association (Declaration of Helsinki), printed in the British Medical Journal (July 18, 1964). 50 patients with breast cancer, 50 patients with benign breast disease, and 49 patients without breast disease were recruited.^18,22^

### Blood Samples

Ten milliliters of venous blood were drawn from each participant using sterile, disposable Vacutainer kits. Blood samples were allowed to clot at room temperature and then centrifuged at 1500 rpm for 5 min. Serum was collected in aliquots of equal volume (500 μL per sample) and stored at −80 °C until use.^18,22^

### Cell Culture of the T47D Cell Line

The breast cancer cell line T47D (ATCC; Manassas, VA, USA) was cultured in phenol-free RPMI 1640 medium supplemented with 10% fetal bovine serum, 100 U/ml penicillin, 100 mU/ml streptomycin, and 250 ng/mL amphotericin B in plastic culture plates (Costar, Cambridge, UK) under atmospheric conditions of 95% humidity and 5% CO2 at 37 °C. When cells reached the desired confluence (85–90%), they were harvested by treatment with cold Versene (PBS + 0.02% EDTA) and pelleted by centrifugation at 1500 rpm for 5 min at room temperature. Cell pellets were washed three times with PBS and stored at −80 °C.^18,22^

### T47D Protein Extraction

Cultured T47D cells were used as a source of antigens for immunoblotting. Cell pellets were lysed in buffer containing 4% (w/v) CHAPS (Bio-Rad, Hercules, CA, USA), 7 M urea (SIGMA-Aldrich, St. Louis, MO, USA), 65 mM DTT (Promega, Madison, WI, USA), and a protease inhibitor cocktail (10 μL per 875 μL lysis buffer; Halt, PIERCE) through 5 freeze–thaw cycles. Lysates were centrifuged at 16,000 × g for 10 min at 4 °C; Supernatants were recovered, pooled, aliquoted and stored at −80 °C until further use. Protein concentration was determined using the Bradford assay (Bio-Rad, Hercules, CA, USA).^18,22^

### SDS-PAGE and Western Blot

Protein extracts from T47D cells (100 μg) were electrophoresed using polyacrylamide gels (4–20% TGX Bio-Rad Laboratories, Hercules, CA, USA) to separate proteins by molecular weight, at 80 V for 2 h. After electrophoresis, the separated proteins were electrophoretically transferred to nitrocellulose membranes (High Bond, Amersham Biosciences, Amersham, UK) at 100 V for 1.25 h using a Trans-Blot mini cell (Bio Rad, Hercules, CA, USA). To confirm protein transfer, membranes were reversibly stained using CPTS (copper phthalocyanine tetrasulfonic acid, Sigma-Aldrich) diluted in 12 mM HCl; Membranes were scanned and then destained, followed by blocking with 5% (w/v) skimmed milk (Svelty, Nestlé, Lagos de Moreno, Jalisco, Mexico) diluted in PBS with 0.3% Tween 20 (PBS-T) for 16 h at 4 °C. Each membrane was marked with two pencil lines, one at its upper limit and one at its lower limit, and then cut vertically into 17 to 18 4 mm-wide strips. Strips were individually incubated with a different serum diluted 1:300 in 2 mL PBS-T for 5 h at room temperature.

Serum coded 111 was used as an internal control to match bands between membranes. After thorough washing with PBS-T, bound antibodies were detected by incubating the membrane strips with goat antihuman IgG antibody H+L-HRP (1:2500; Thermo Scientific, Waltham, MA, USA) for 1 h at room temperature. Peroxidase activity was revealed by incubating the membranes with 0.1 mg/mL 3,3′-diaminobenzidine tetrahydrochloride (Sigma-Aldrich, St. Louis, MO, USA) and 0.015% hydrogen peroxide in PBS-T for 5 min at room temperature. The peroxidase reaction was stopped by gently washing the strips five times with deionized water. The strips were allowed to air-dry overnight, protected from light exposure. Serum 111 is a normal sample from a 47-year-old woman. It was chosen because the strip showed bands across the molecular weight range.^18,22^

### Western Blot Images

**Figure 1:**
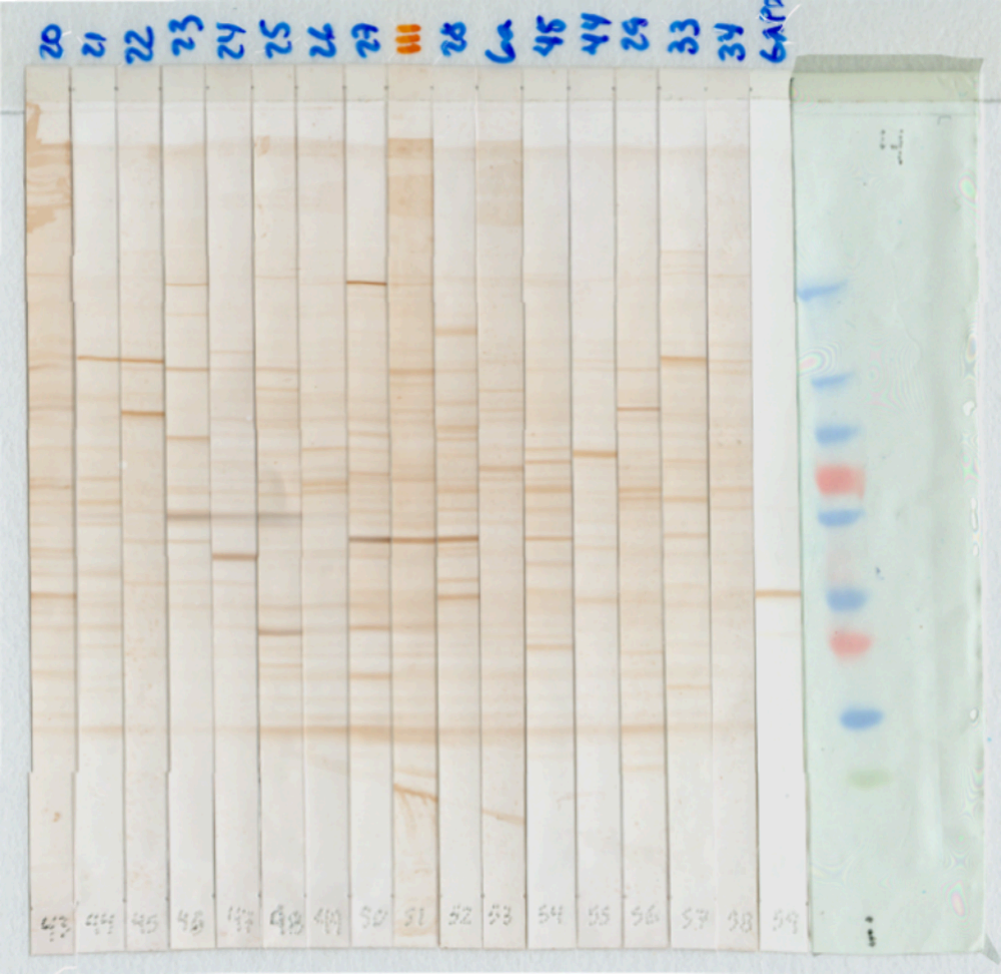
**Western Blot image.** Image containing the nitrocellulose membrane strips with the expression of bands obtained from the Western Blot of the total protein lysate of the T47D cell line (ductal carcinoma of the breast). The blue numbers at the top of the strips are the patient identifiers. The red number at the top of the strips is the control patient. The gray numbers at the bottom of the strips indicate the sequence in which the strips were arranged to form the image. The strip on the left is the molecular weight marker. The blue bands represent molecular weights from 10 kDa to 250 kDa, pink from 25 kDa to 75 kDa, green from 37 kDa.

The strips were scanned (Hewlett-Packard Scanjet G4050, Böblingen, Germany) in TIF format at a resolution of 300 dpi; all images were obtained with the same hue, saturation and intensity settings. Once captured, the strips were aligned in Adobe Photoshop CS5 using the pencil lines drawn on the membrane as a reference, thus creating images containing an average of 16 strips from the 149 patients (cancer, benign breast pathology, no breast pathology), obtaining 15 color images in TIF format (Figure 1).^18,22^

### Classification of Proteomic Images Using Supervised Learning

**Figure 2:**
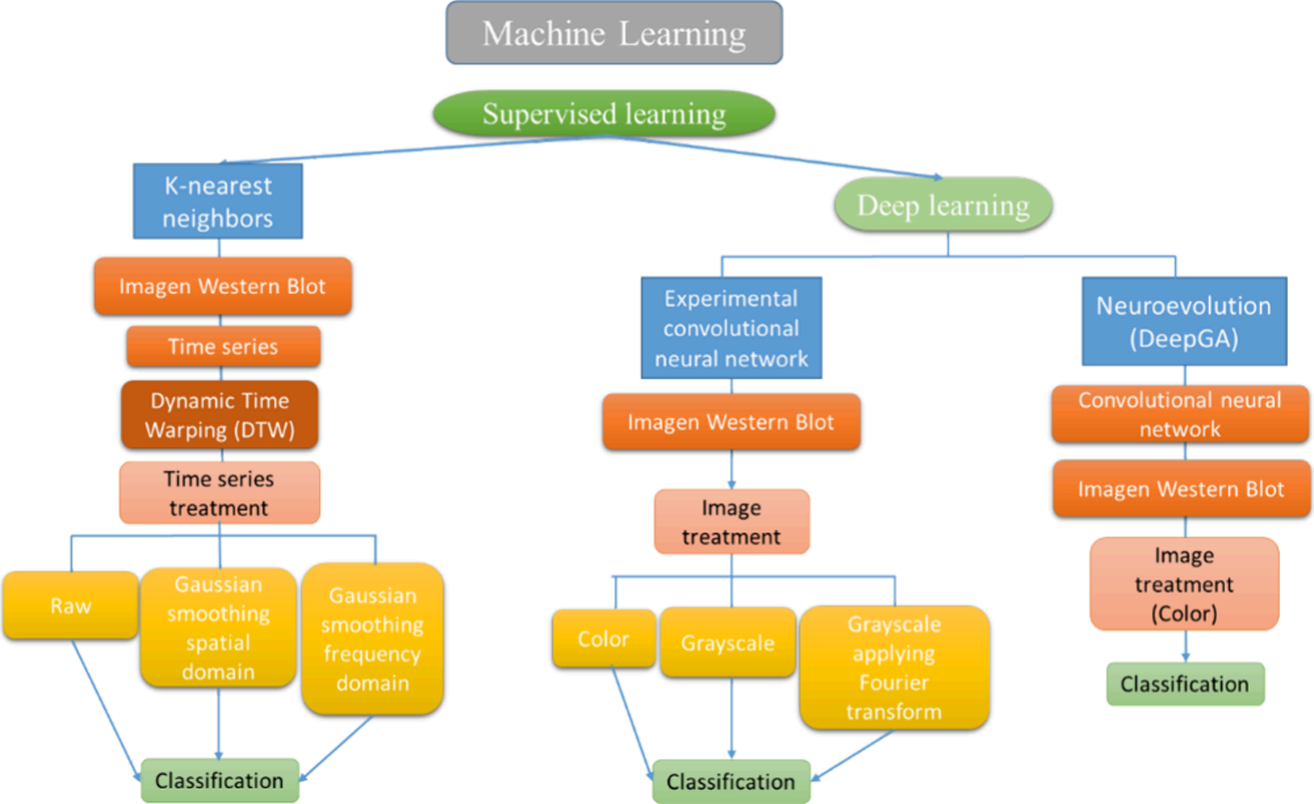
**Classification techniques.** Diagram of the proposed techniques for the classification of Western Blot images. The diagram is arranged in the form of colored blocks that describe the processes in each of them. ■ It represents the classification techniques used. ■ Use of the image (time series/image/classification technique). ■ Similarity measure. ■Time series treatment.

In this work, proteomic images were analyzed and classified to discriminate between patients without breast pathology, benign breast pathology and patients with malignant breast pathology, for the early detection of breast cancer, using supervised learning techniques (subarea of machine learning) such as the K-nearest neighbors algorithm in conjunction with the Dynamic Time Warping (DTW) technique as a similarity measure and convolutional neural networks obtained both experimentally and through neuroevolution (Figure 2).

Machine learning and deep learning techniques, which were explored to classify banding patterns in proteomic images, were:

- K-Nearest Neighbors (KNN) in conjunction with Dynamic Time Warping (DTW) as a similarity measure.
- Experimental Convolutional Neural Network (ECNN)
- Convolutional Network obtained with Neuroevolution using the DeepGA algorithm (CNN-DeepGA)

These techniques were evaluated using the Holdout method. Which consists of dividing the data set (in our case time series/images) into a training set and a test set. The classification model is built with the training set, and its performance is evaluated with the test data.^23^ In this work, to evaluate the performance of the classifiers, 70% of the data was used for training and 30% for testing.

### K-Nearest Neighbors (KNN)

**Figure 3:**
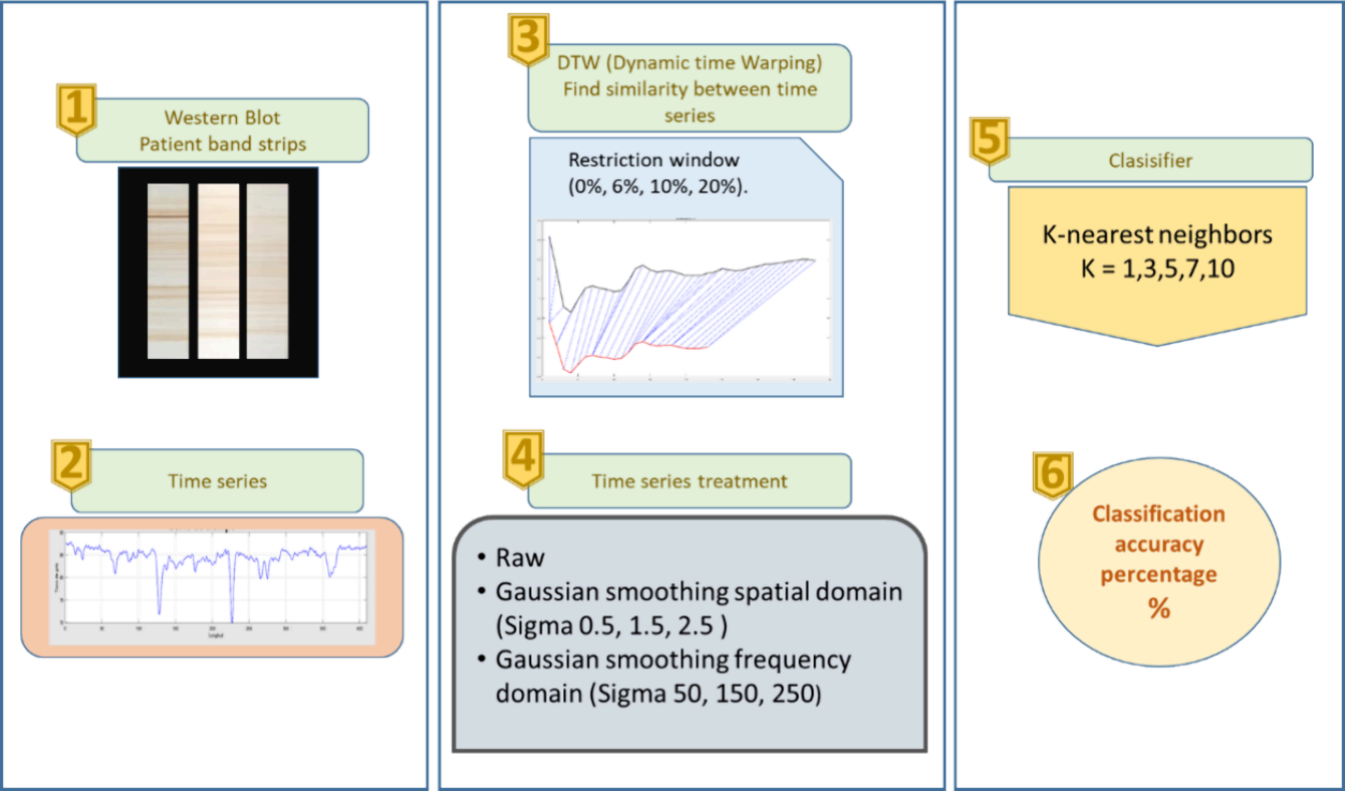
**K-Nearest Neighbors Classification.** Time series classification scheme using the K-Nearest Neighbors algorithm, Dynamic Time Warping (DTW) similarity distance, and time series treatments.

The K-nearest neighbors algorithm is a supervised learning classification technique. The objective of this algorithm is to classify a new example in the class of its k closest neighbors.^23^ The K values used for this work were 1,3,5,7,10. Ten runs were carried out, obtaining an average of the percentage of classification accuracy in each of them. The examples used for classification are represented by time series; the proposed scheme to carry out this procedure is shown in Figure 3.

#### Time Series

**Figure 4:**
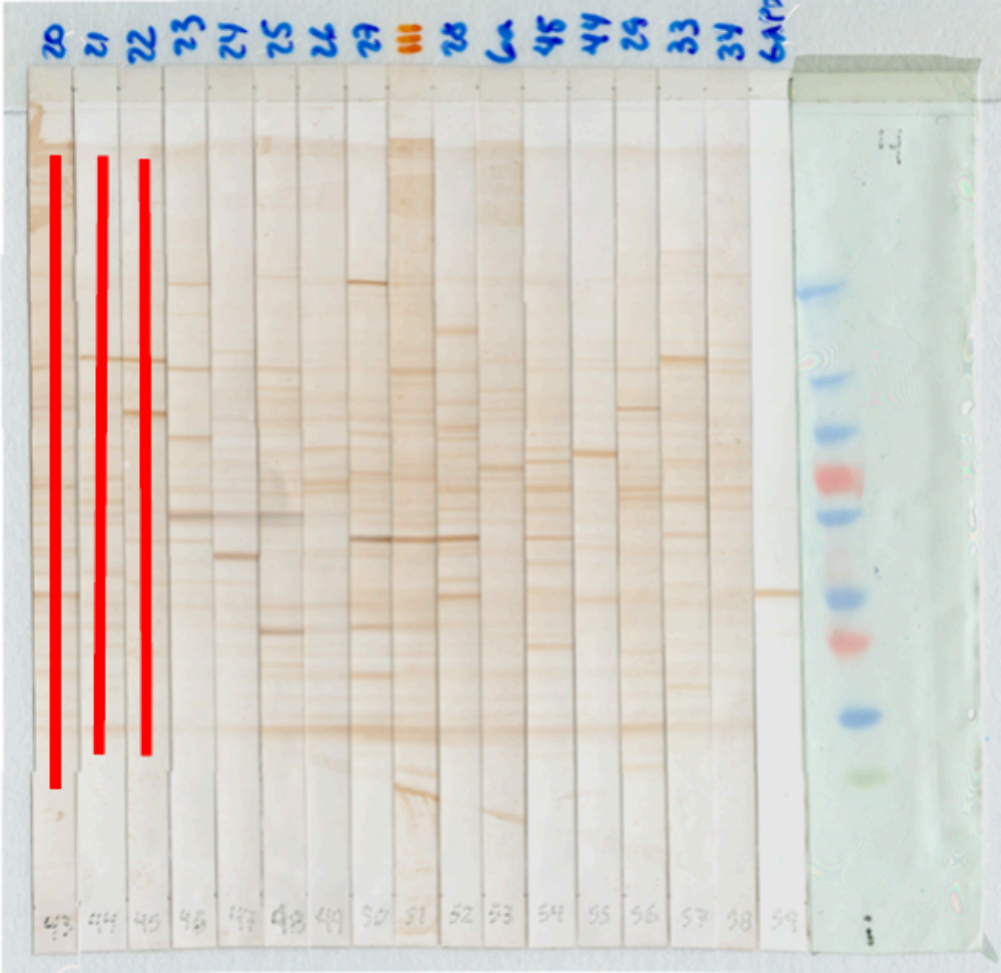
**Obtaining time series.** Image with an average of 16 strips containing the antibody–antigen reaction bands of patients. The red lines represent the selection of the area to obtain the time series. The area was chosen manually.

The time series were formed by the intensity value of the pixels that make up each band of the nitrocellulose strips. The time series were obtained manually by delimiting an area of the banding pattern on each strip. The length of the time series is determined by the number of pixels that make up the chosen area. The algorithm with which the chosen area is manually obtained internally converts it to grayscale, thereby acquiring the value of the intensity of the pixels. The intensity is what determines the variability of the time series. The time series have dissimilar lengths as shown in Figure 4.

#### Similarity Measure between Time Series

DTW (Dynamic Time Warping) was used, which allows obtaining a measure of similarity between time series of dissimilar lengths. Unlike Euclidean distance that compares two-time series point to point, DTW compares them from one to many (and vice versa).^24^ One problem that this method can have is the assignment of a large number of points from one-time series (A) to a single point from another time series (B). To avoid this drawback, the similarity search space between two-time series is restricted using restriction windows.^25^ The restriction values used in this work were 6%, 10% and 20%, which are based on the length of the longest time series.^26^ On the other hand, the similarity search was also carried out between time series without a restriction window, represented with the value of 0.

#### Time Series Treatment

The time series were treated in three ways: 1) Raw values, 2) Gaussian smoothing in the space domain, 3) Gaussian smoothing in the frequency domain. The two types of Gaussian smoothing are low-pass filters that allow the noise that an image has to be reduced and in our case, it is expected to increase the performance of the classifier. Gaussian smoothing in the space domain is applied directly to the image pixels (time series), through a convolution (multiplication) of a mask (Kernel) with predefined NxM dimension values. For Gaussian smoothing in the frequency domain, we pass from the space domain to the frequency domain using the Fourier transform, to apply the Gaussian filter and pass only the low frequencies that allow the smoothing of the time series.^27^ In this type of filters the sigma value determines the level of smoothing. For Gaussian smoothing in the space domain, the chosen sigma values were 0.5, 1.5, 2.5. For Gaussian smoothing in the frequency domain, the sigma values were 50, 150, 250.

### Experimental Convolutional Neural Network (ECNN)

In the area of deep learning, convolutional neural networks (CNN) are effective tools in the interpretation of medical images in applications such as the detection and classification of benign and malignant tumors, detection of colon cancer, breast cancer, heart anomalies, etc.^28^

**Figure 5:**
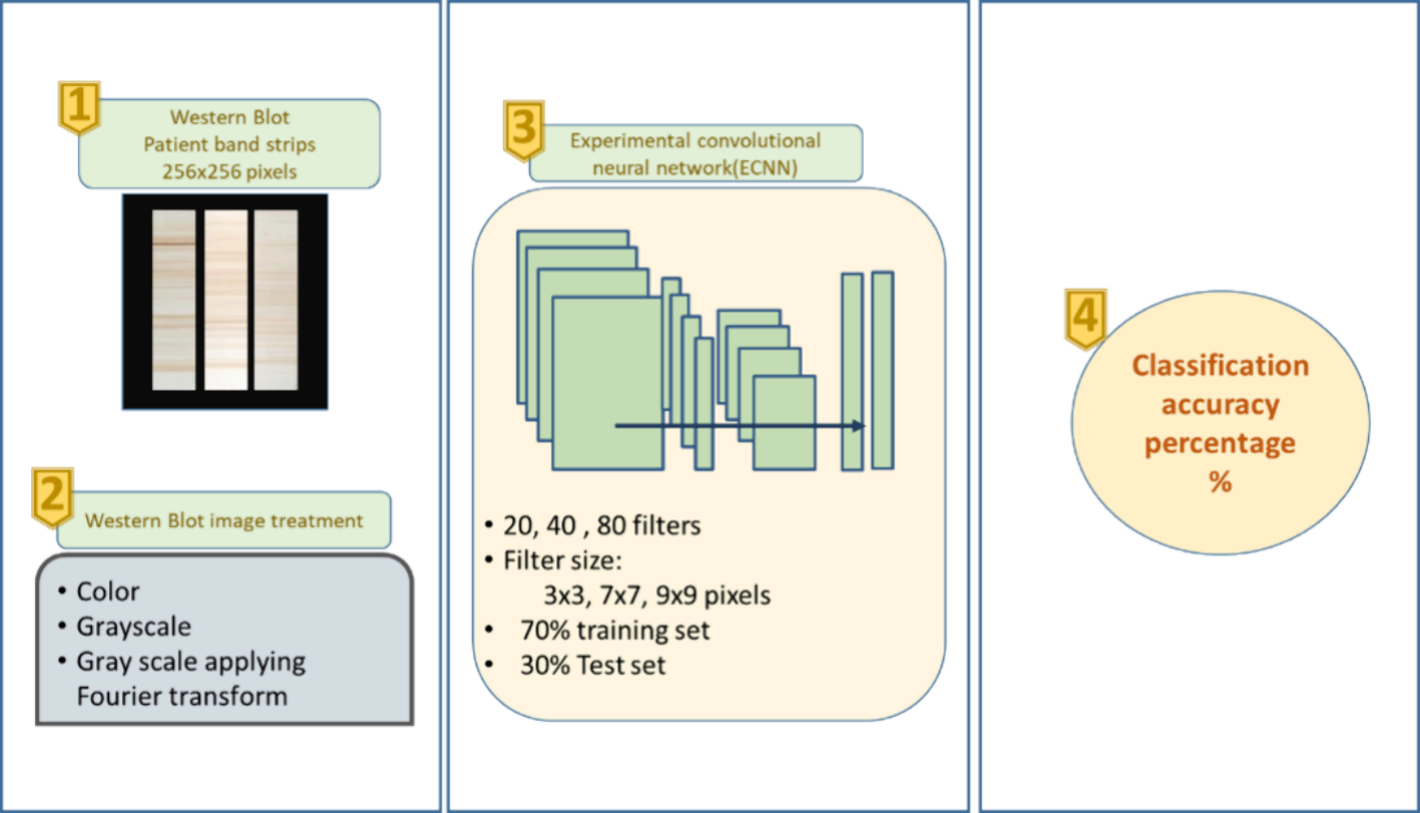
**Classification with ECNN.** Western Blot image classification scheme using experimental Convolutional Neural Networks (ECNN) and different image treatments.

A convolutional neural network (CNN) is an artificial neural network that uses machine learning to analyze images, classify visual elements, and perform computer vision tasks. In CNN is necessary to find an ideal architecture for the data being analyzed, and this is done by trial and error, which consumes a lot of time and this does not guarantee good performance in the classification.^29^Figure 5 shows the scheme for using an Experimental CNN (ECNN) for Western Blot image classification, previously proposed.^19^

#### Western Blot Strip Image Treatment

The images were treated in 3 ways: 1) Color, with dimensions of 256 × 256 pixels, 2) Gray scale, with dimensions of 256 × 256 pixels, 3) Gray scale, using the descriptors obtained from the application of the Fourier transform, having an image dimension of 256 × 15 pixels. The images have had to be sized to 256 × 256 pixels due to the RNCE implementation specification.

#### Architecture of the Experimental Convolutional Neural Network

**Figure 6:**
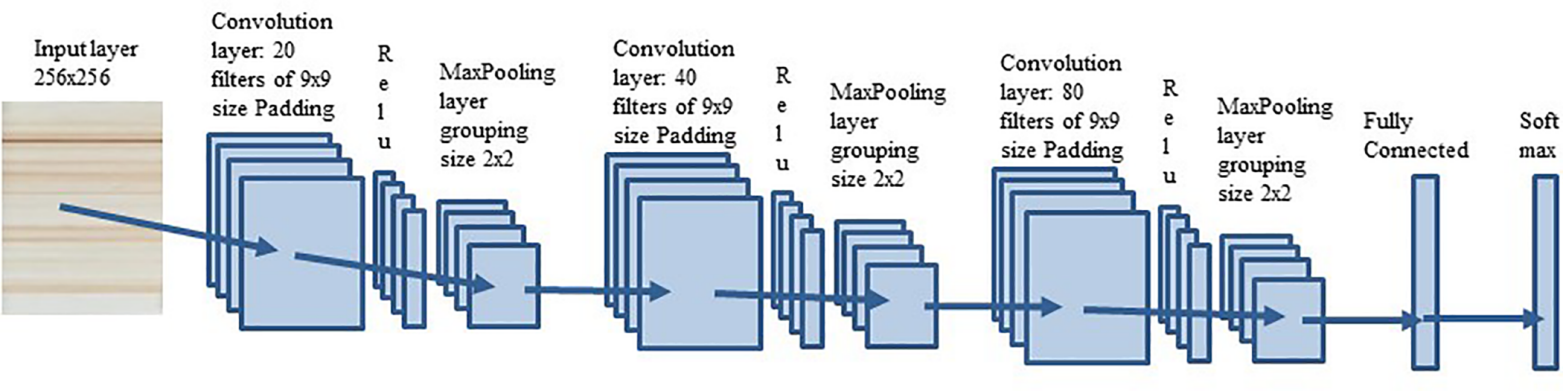
**ECNN architecture.** Experimentally obtained CNN architecture used in this work for Western Blot image classification.

In this work, the ECNN architecture was designed by iteratively and manually adjusting the number and type of hidden layers, as well as the parameters of each of them. The architecture that was obtained experimentally had the following characteristics and is shown graphically in Figure 6:

1. Input layer: 256 × 256
2. Convolution layer: 20 filters of 9 × 9 size Padding
3. Normalization Layer/ReLu Layer
4. maxPooling layer - grouping size 2 × 2
5. Convolution layer: 40 filters of 9 × 9 size Padding
6. Normalization Layer/ReLu Layer
7. maxPooling layer - grouping size 2 × 2
8. Convolution layer: 80 filters of 9 × 9 size Padding
9. Normalization Layer/ReLu Layer
10. Fully connected layer
11. Classification layer with softmax method for values normalization

To train the network, 20 epochs (epoch - is a complete training run of a model on a data set) were run on the color and grayscale images. In the case of the gray scale images applying the Fourier transform, 70 epochs were tested, because in this number of epochs the network converged. In the case of convolution kernels, the kernel sizes used were 3, 7, and 9 coefficients. Ten runs were carried out, obtaining an average of the classification accuracy in each of them.

Being a first approach to this model, the objective was to find an ideal Convolutional Neural Network architecture according to the number of images available, for this reason artificial data augmentation was not considered.

### Convolutional Neural Network Obtained with Neuroevolution Using the DeepGA Algorithm

**Figure 7:**
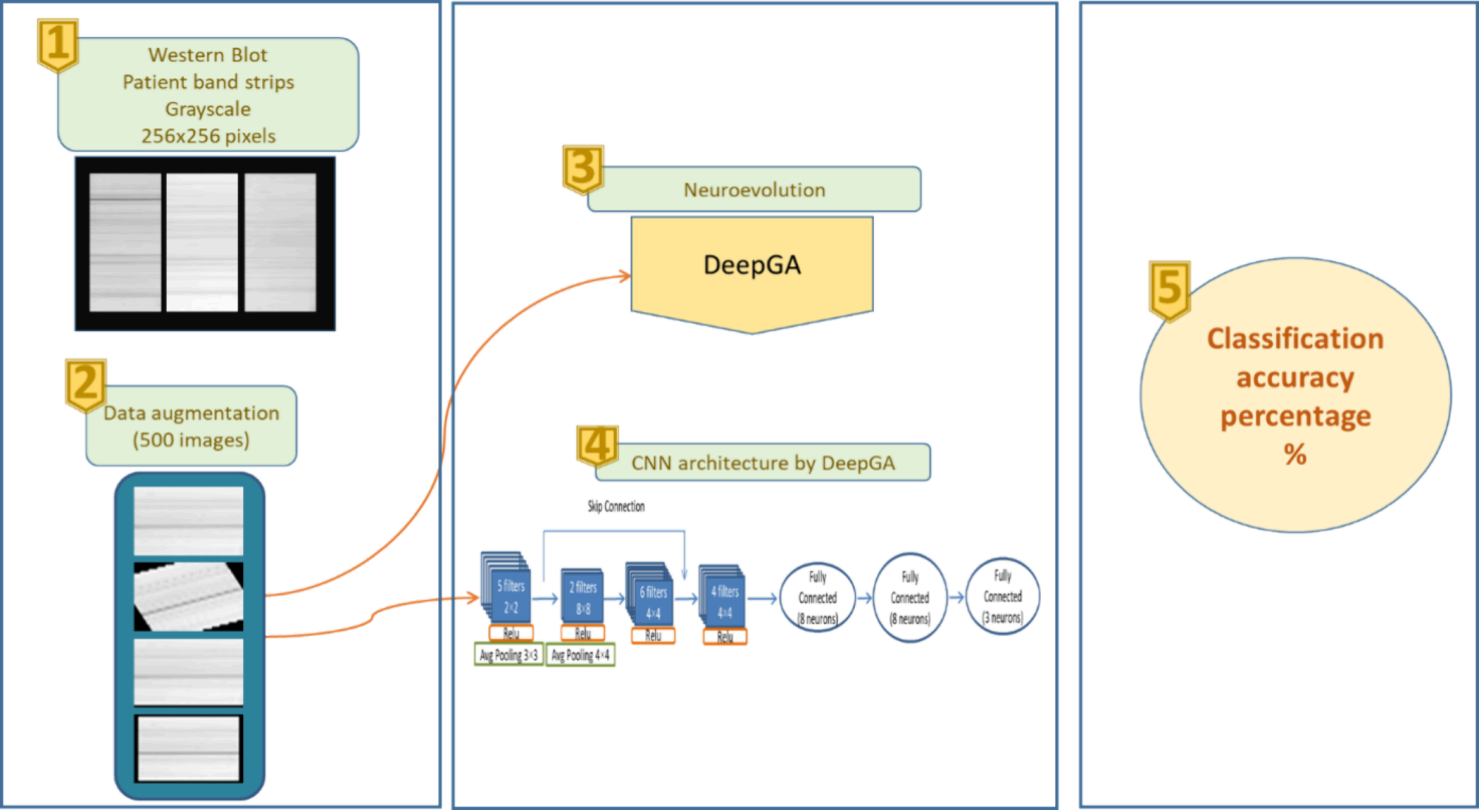
**Classification with DeepGA.** Western Blot image classification scheme using the DeepGA neuroevolution algorithm.

Figure 7 shows the diagram that specifies the process carried out to obtain a convolutional neural network using the DeepGA neuroevolution algorithm,^20^ and which was previously used to analyze and classify Western Blot images of women with and without breast pathology.^21^

#### Western Blot Strip Image Treatment

The images were used in gray scale with dimensions of 256 × 256 pixels.

#### Data Augmentation

One of the problems that arises in learning algorithms is the management of biases such as overfitting and underfitting, in addition to the fact that in the medical area it is most of the time difficult to have large volumes of images. To solve these problems, data augmentation was carried out, artificially increasing the original volume of the images five times for each class of patient, going from 49 (for a patient without breast pathology) or 50 (for the other two classes). of patients), to 250 images in each class, achieving a database of 750 images. With this, it was possible to have a base of balanced images, of which 250 belong to the class of patients with breast cancer, 250 to the class of patients with benign breast pathology and 250 to the class of patients without breast pathology.

To increase the data, affine transformations were used randomly and with a range of degrees, movement or size, which are 1) Rotation, with a range of degrees from 10 to 30, 2) translation with a range of movement of 0.1 to 0.3, 3) scaling with a size range of 0.5 to 1, 4) Gaussian blur, with a kernel size of 7.

#### Neuroevolution to Data (DeepGA)

To obtain the ideal architecture for the images using the DeepGA neuroevolution algorithm, the first step was to adjust the algorithm parameters which are shown in Table 1.

**Table 1:** Parameters used in DeepGA^a^

parameters	values
population size	20
number of generations	50
crossover rate	0.7
mutation rate	0.5
tournament size	4

a List of parameters used in DeepGA for generating an CNN architecture suitable for Western Blot image classification.

**Figure 8:**
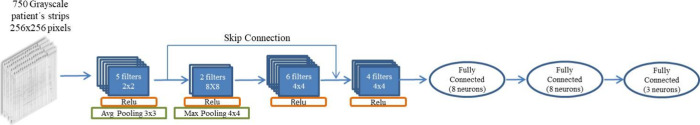
**CNN architecture obtained by DeepGA.** The CNN consists of 4 convolutional layers of 5, 2, 6, 4 filters of different sizes, as well as 3 fully connected layers of 8 and 3 neurons.

The parameters proposed in Vargas-Hákim et al. (2021)^20^ were taken as a basis and adjusted according to the results obtained in a series of experiments that were carried out with Western Blot images. One of the objectives of using DeepGA, in addition to obtaining an RNC topology, was to maintain a feasible execution time with the availability of the resources available. The best topology obtained by DeepGA is shown in Figure 8.^21^

#### Classification of Western Blot Images Using CNN Obtained by DeepGA

To perform the classification of Western Blot strip images, using a convolutional neural network (CNN) obtained by neuroevolution, the following process was carried out:

The CNN originated using the DeepGA neuroevolution algorithm (CNN-DeepGA) was trained, taking as input data the database of 750 grayscale images of Western Blot strips. The training of CNN-DeepGA consisted of only 10 epochs (Sun et al., 2020). For training, 70% of the data set (525 images out of 750 in total) was used, while 30% of the main set was used for testing (225 images out of 750 in total). To evaluate the performance of CNN-DeepGA, 10 runs were carried out, obtaining an average of the percentage of classification accuracy in each of them.

Additional material and algorithm code can be reviewed at:

https://github.com/jllagunoroque/WesternBlot_Classification

## Results

Two experiments were carried out based on the number of classes, varying the data treatment in each of them. The first experiment was done with three classes (patients with breast cancer, with benign breast pathology and without benign pathology) and the second with two classes (patients with breast cancer, without benign pathology).

With the results obtained, the accuracy, sensitivity, specificity and precision of the classifiers were calculated.

Accuracy was calculated from the total number of predictions that the algorithm correctly classified divided by the total number in the data set (eq 1).
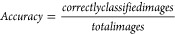
1

Sensitivity is the number of items correctly identified as positive out of the total number of true positives (eq 2).
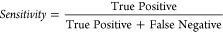
2

Specificity is the number of items correctly identified as negative out of the total number of negatives (eq 3).
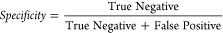
3

Precision is the number of items correctly identified as positive out of a total of items identified as positive (eq 4).
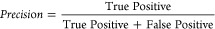
4

### K-Nearest Neighbors (KNN)

#### Experiments with Three Classes

The experiments were carried out taking into account three classes: patients with breast cancer, with benign breast pathology and without benign breast pathology, as well as the treatments to the time series and the variations to the values of the restriction windows of the DTW technique (Table 2).

**Table 2:** Classification of 3-Class Time Series: Comparison of the Best Results of Classifying Time Series with Different Treatments Using the K-Nearest Neighbors Algorithm^a^

treatment	accuracy %	precision %	sensitivity %	specificity %
GFCR3K520150	74.67	75.20	74.67	87.33
GFSR3K350	72.00	72.05	72.00	86.00
GESR3K1025	63.56	63.51	63.56	81.78
RCR3K506	63.56	63.27	63.56	81.78
GECR3K100615	63.33	63.98	63.33	81.67
RSR3K5	59.78	59.82	59.78	79.89

a **Nomenclature of each of the treatments** GFCR3K520150 Gaussian Frequencies with Restrictions 3 classes K = 5 Window = 0.20 sigma = 150. GFSR3K350 Gaussian Frequencies without Restrictions 3 classes K = 3 sigma = 50. GESR3K1025 Gaussian Spatial without Restrictions 3 classes K = 10 sigma = 2.5. RCR3K506 Raw with Restrictions 3 classes K = 5 windows = 0.06. GECR3K100615 Gaussian Spatial with Restrictions 3 classes K = 10 window = 0.6 sigma = 1.5. RSR3K5 Raw without Restrictions 3 classes K = 5.

#### Experiments with Two Classes

The experiments were carried out taking into account two classes: patients with breast cancer and without benign breast pathology, as well as the treatments to the time series and the variations to the values of the restriction windows of the DTW technique.

Table 3 shows the best results for each treatment. The best result for the treatment of raw data without restrictions was obtained with RSR2K5 with 78.67% accuracy, precision of 82.23%, sensitivity of 78.67%, specificity of 82.00%. The best classification result for raw data with restrictions was obtained with RCR2K1010 with 84.00% accuracy, precision of 81.59%, sensitivity of 84.00%, specificity of 80.67%.

**Table 3:** Classification of 2-Class Time Series: Comparison of the Best Results of Classifying Time Series with Different Treatments Using the K-Nearest Neighbors Algorithm^a^

treatment	accuracy %	precision %	sensitivity %	specificity %
GFCR2K1010150	96.67	76.35	96.67	69.33
GFSR2K10250	92.67	74.91	92.67	68.00
GECR2K70625	90.00	87.63	90.00	86.00
RCR2K1010	84.00	81.59	84.00	80.67
GESR2K715	80.67	76.63	80.67	74.00
RSR2K5	78.67	82.23	78.67	82.00

a **Nomenclature of each of the treatments.** GFCR2K1010150 Gaussian Frequencies with Restrictions 2 classes K = 10 window = 0.10 sigma = 150. GFSR2K10250 Gaussian Frequencies without Restrictions 2 classes K = 10 sigma = 250. GECR2K70625 Gaussian Spatial with Restrictions 2 classes K = 7 window = 0.06 sigma = 2.5. RCR2K1010 Raw with Restrictions 2 classes K = 10 windows = 0.10. GESR2K715 Gaussian Spatial without Restrictions 2 classes K = 7 sigma = 1.5. RSR2K5 Raw without Restrictions 2 classes K = 5.

The best classification result with Gaussian smoothing in space domain with restrictions was obtained with GESR2K715 with 80.67% accuracy, precision 76.63%, sensitivity 80.67%, specificity 74.00%. The best Gaussian smoothing classification result in space domain with restrictions was obtained with GECR2K70625 with 90.00% accuracy, 87.63% precision, 86.00% sensitivity, and 86.00% specificity.

The best Gaussian smoothed classification result in frequency domain without restrictions was obtained with GFSR2K10250 with 92.67% accuracy, 74.91% precision, 92.67% sensitivity, 68.00% specificity. The best Gaussian smoothed classification result in the frequency domain with restrictions was obtained with GFCR2K1010150 with 96.67% accuracy, 76.35% precision, 96.67% sensitivity, 69.33% specificity.

### Experimental Convolutional Neural Network (ECNN)

#### Experiments with Three Classes

The experiments were carried out taking into account three classes: patients with breast cancer, with benign breast pathology and without benign breast pathology, as well as the treatments to the images and the different sizes of convolution kernels.^19^

Table 4 shows the results for each treatment. The best result for the treatment of color images was obtained with ECNN3CColor3 × 3 with 64.45% accuracy, precision of 64.15%, sensitivity of 64.44%, specificity of 82.20%. The best result for the treatment of gray scale images was obtained with ECNN3CGray7 × 7 with 66.89% accuracy, precision of 67.27%, sensitivity of 66.89%, specificity of 83.44%. The best result for the treatment of gray scale images, using the descriptors obtained from the application of the Fourier transform, was obtained with ECNN3CFourier9 × 9 with 61.56% accuracy, precision of 61.56%, sensitivity of 61.56%, specificity of 80.78%.

**Table 4:** ECNN Classification for 3 Classes: Comparison of the Results of Classifying Western Blot Image Strips with ECNN^a^

treatment	accuracy %	precision %	sensitivity %	specificity %
ECNN3CGray7 × 7	66.89	67.27	66.89	83.44
ECNN3CColor3 × 3	64.45	64.15	64.44	82.20
ECNN3CGray3 × 3	62.89	64.56	62.89	81.44
ECNN3CFourier9 × 9	61.56	61.14	61.56	80.78
ECNN3CColor9 × 9	61.55	61.95	61.56	80.78
ECNN3CFourier3 × 3	61.11	60.72	61.11	80.56
ECNN3CGray9 × 9	60.67	60.74	60.67	80.33
ECNN3CFourier7 × 7	59.56	59.37	59.56	79.78
ECNN3CColor7 × 7	58.22	58.81	58.22	79.11

a **Nomenclature of each of the treatments.** ECNN3CGray7 × 7 Experimental Convolutional Neural Network 3 Classes gray scale 7 × 7. ECNN3CColor3 × 3 Experimental Convolutional Neural Network 3 Classes Color 3 × 3. ECNN3CGray3 × 3 Experimental Convolutional Neural Network 3 Classes gray scale 3 × 3. ECNN3CFourier9 × 9 Experimental Convolutional Neural Network 3 Classes gray scale applying Fourier transform 9 × 9. ECNN3CColor9 × 9 Experimental Convolutional Neural Network 3 Classes Color 9 × 9. ECNN3CFourier3 × 3 Experimental Convolutional Neural Network 3 Classes gray scale applying Fourier transform 3 × 3. ECNN3CGray9 × 9 Experimental Convolutional Neural Network 3 Classes gray scale 9 × 9. ECNN3CFourier7 × 7 Experimental Convolutional Neural Network 3 Classes gray scale applying Fourier transform 7 × 7. ECNN3CColor7 × 7 Experimental Convolutional Neural Network 3 Classes Color 7 × 7.

#### Experiments with Two Classes

The experiments were carried out taking into account two classes: patients with breast cancer, and without benign breast pathology, as well as the treatments to the images and the different sizes of convolution kernels.^19^

Table 5 shows the results for each treatment. The best result for the treatment of color images was obtained with ECNN2CColor3 × 3 with 80.67% accuracy, precision of 80.26%, sensitivity of 81.33%, specificity of 80.00%. The best result for the treatment of gray scale images was obtained with ECNN2CGris3 × 3 with 82.33% accuracy, precision of 83.92%, sensitivity of 80.00%, specificity of 84.67%. The best result for the treatment of gray scale images, using the descriptors obtained from the application of the Fourier transform, was obtained with ECNN2CFourier3 × 3 with 86.00% accuracy, precision of 85.06%, sensitivity of 87.33%, specificity of 84.67%.

**Table 5:** ECNN Classification for 2 Classes: Comparison of the Results of Classifying Western Blot Image Strips with ECNN^a^

treatment	accuracy %	precision %	sensitivity %	specificity %
ECNN2CFourier3 × 3	86.00	85.06	87.33	84.67
ECNN2CGris3 × 3	82.33	83.92	80.00	84.67
ECNN2CFourier7 × 7	81.00	80.00	82.67	79.33
ECNN2CFourier9 × 9	81.00	78.18	86.00	76.00
ECNN2CColor3 × 3	80.67	80.26	81.33	80.00
ECNN2CColor9 × 9	80.00	78.85	82.00	78.00
ECNN2CColor7 × 7	79.33	77.50	82.67	76.00
ECNN2CGris7 × 7	77.00	79.56	72.67	81.33
ECNN2CGris9 × 9	76.33	77.62	74.00	78.67

a **Nomenclature of each of the treatments.** ECNN2CFourier3 × 3 Experimental Convolutional Neural Network 2 Classes gray scale applying Fourier transform 3 × 3. ECNN2CGris3 × 3 Experimental Convolutional Neural Network 2 Classes gray scale 3 × 3. ECNN2CFourier7 × 7 Experimental Convolutional Neural Network 2 Classes gray scale applying Fourier transform 7 × 7. ECNN2CFourier9 × 9 Experimental Convolutional Neural Network 2 Classes gray scale applying Fourier transform 9 × 9. ECNN2CColor3 × 3 Experimental Convolutional Neural Network 2 Classes Color 3 × 3. ECNN2CColor9 × 9 Experimental Convolutional Neural Network 2 Classes Color 9 × 9. ECNN2CColor7 × 7 Experimental Convolutional Neural Network 2 Classes Color 7 × 7. ECNN2CGris7 × 7 Experimental Convolutional Neural Network 2 Classes gray scale 7 × 7. ECNN2CGris9 × 9 Experimental Convolutional Neural Network 2 Classes gray scale 9 × 9.

### Convolutional Network Obtained with DeepGA

#### Experiments with Three Classes

The experiments were carried out taking into account three classes: patients with breast cancer, with benign breast pathology and without benign breast pathology. Grayscale images were used so it is the only treatment for the images.^21^

Table 6 shows the classification results of the CNN obtained using the DeepGA neuroevolution algorithm for three classes. The values obtained were 90.67% accuracy, 90.96% precision, 90.71% sensitivity and 95.34% specificity.

**Table 6:** CNN-DeepGA Classification with 3 Classes: Results of Classification of Western Blot Strip Images with Three Classes Using a Convolutional Neural Network Obtained by Neuroevolution (DeepGA)^a^

treatment	accuracy %	precision %	sensitivity %	specificity %
CNN3-DeepGA	90.67	90.96	90.71	95.34

a These results were previously reported.^21^

#### Experiments with Two Classes

The experiments were carried out taking into account two classes: patients with breast cancer, and without benign breast pathology. Grayscale images were used so it is the only treatment for the images. These experiments are a proposal of this study.

Table 7 shows the classification results of the CNN obtained using the DeepGA neuroevolution algorithm for two classes. The values obtained were 95.13% accuracy, 96.65% precision, 93.39% sensitivity and 96.84% specificity.

**Table 7:** CNN-DeepGA Classification with 2 Classes: Results of Classification of Western Blot Strip Images with Three Classes Using a Convolutional Neural Network Obtained by Neuroevolution (DeepGA)^a^

treatment	accuracy %	precision %	sensitivity %	specificity %
CNN2-DeepGA	95.13	96.65	93.39	96.84

a These are results of the experiments proposed in this study.

### Comparison of Classification Performance between the Methods Proposed in This Work

The comparison of the classification results of the image treatment by each of the methods proposed in this work was carried out, with the objective of showing the method that has the best classification performance. This comparison was made for two and three classes.

#### Comparison of Results for Three Classes

The experiments were carried out taking into account three classes: patients with breast cancer, with benign breast pathology and without benign breast pathology.

Table 8 shows the best classification results of image processing by each of the proposed methods for three classes. The three best classification results correspond in first place to CNN3-DeepGA with 90.67% accuracy, precision of 90.96%, sensitivity of 90.71%, specificity of 95.34%. Second place goes to GFCR3K520150 with 74.67% accuracy, 75.2% precision, 74.67% sensitivity, 87.33% specificity. Third place is for ECNN3CGray7 × 7 with 66.89% accuracy, precision of 67.27%, sensitivity of 66.89%, specificity of 83.44%.

**Table 8:** Classification of Proposed Methods for 3 Classes: Comparison of Classification Results of Image Processing by Each of the Methods Proposed in This Work^a^

treatment	accuracy %	precision %	sensitivity %	specificity %
*CNN3-DeepGA	90.67	90.96	90.71	95.34
***GFCR3K520150	74.67	75.2	74.67	87.33
**ECNN3CGray7 × 7	66.89	67.27	66.89	83.44
**ECNN3CColor3 × 3	64.45	64.15	64.44	82.20
***GESR3K1025	63.56	63.51	63.56	81.78
***RCR3K506	63.56	63.27	63.56	81.78
**ECNN3CFourier9 × 9	61.56	61.14	61.56	80.78

a **Image/time series processing methods used.** *Convolutional neural network obtained by neuroevolution (DeepGA). **Experimental Convolutional Neural Network. ***Time series treatment. Nomenclature of each of the treatments. *CNN3-DeepGA Convolutional neural network obtained by neuroevolution (DeepGA) 3 classes. ***GFCR3K520150 Gaussian Frequencies with Restrictions 3 classes K = 5 Window = 0.20 sigma = 150. **ECNN3CGris7 × 7 Experimental Convolutional Neural Network 3 Classes gray scale 7 × 7. **ECNN3CColor3 × 3 Experimental Convolutional Neural Network 3 Classes Color 3 × 3. ***GESR3K1025 Gaussian Spatial without Restrictions 3 classes K = 10 sigma = 2.5. ***RCR3K506 Raw with Restrictions 3 classes K = 5 windows = 0.06. **ECNN3CFourier9 × 9 Experimental Convolutional Neural Network 3 Classes gray scale applying Fourier transform 9 × 9.

**Figure 9:**
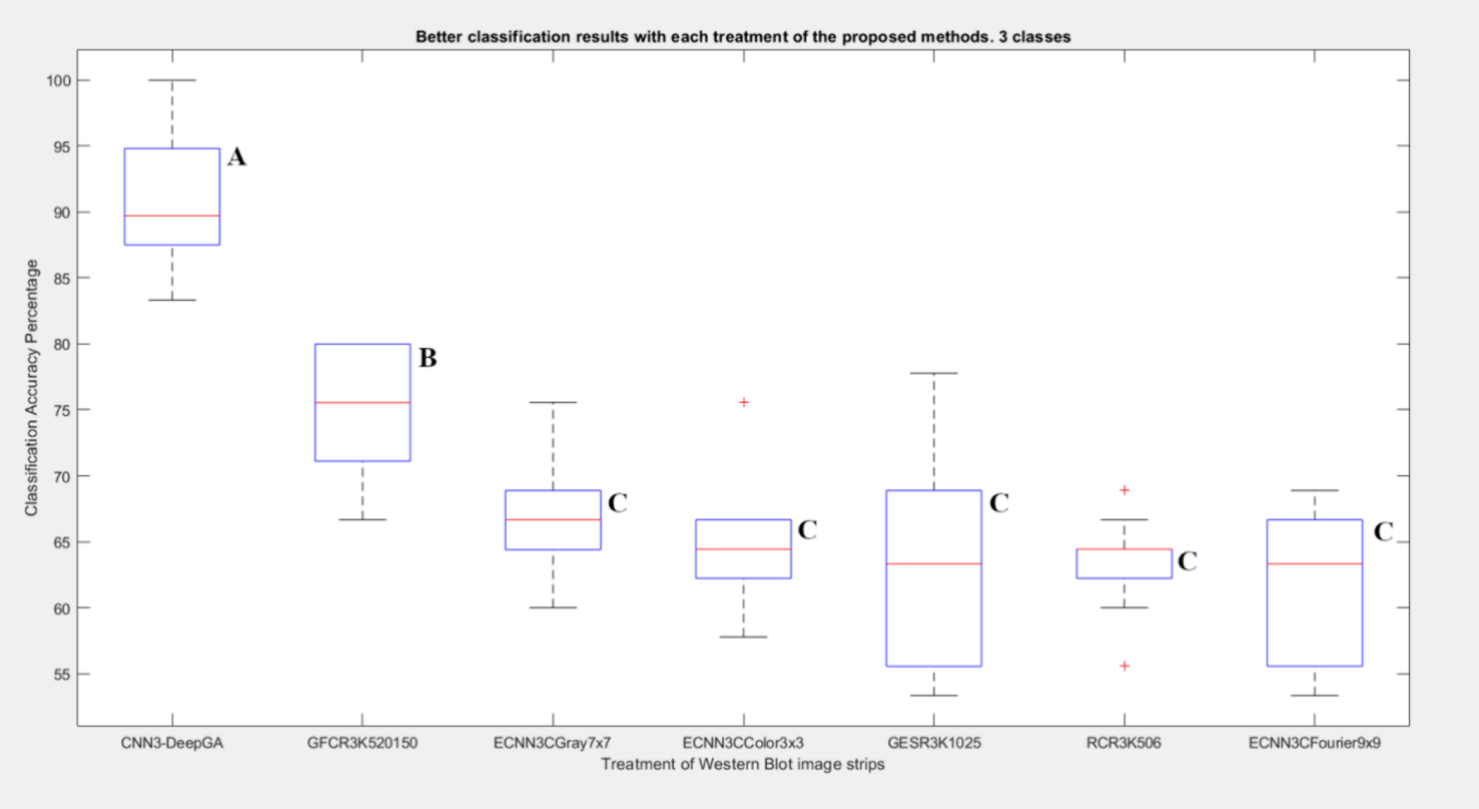
**Variability of classification of proposed methods for 3 classes.** The variability of classifications with the proposed methods with the different image treatments is shown with respect to accuracy. Box plots that do not share a letter are significantly different.

As shown in Figure 9, ECNN3CGray7 × 7 and ECNN3CColor3 × 3 showed more consistent results due to low variability in classification accuracy values, while GESR3K1025 showed greater dispersion in classification accuracy values. However, CNN3-DeepGA had the highest performance and showed significant differences from the rest of the methods, regarding accuracy (*p* < 0.05).

#### Comparison of Results for Two Classes

The experiments were carried out taking into account two classes: patients with breast cancer and without benign breast pathology.

Table 9 shows the best classification results of image processing by each of the proposed methods for two classes. The top three classification results correspond first to CNN2-DeepGA with 95.13% accuracy, 96.65% precision, 93.39% sensitivity, 96.84% specificity. Second place goes to GFCR2K1010150 with 96.67% accuracy, 76.35% precision, 96.67% sensitivity, 69.33% specificity. Third place is for GECR2K70625 with 90.00% accuracy, 87.62% precision, 90.00% sensitivity, 86.00% specificity.

**Table 9:** Classification of Proposed Methods for 2 Classes: Comparison of Classification Results of Image Processing by Each of the Methods Proposed in This Work^a^

treatment	accuracy %	precision %	sensitivity %	specificity %
*CNN2-DeepGA	95.13	96.65	93.39	96.84
***GFCR2K1010150	96.67	76.35	96.67	69.33
***GECR2K70625	90.00	87.62	90.00	86.00
**ECNN2CFourier3 × 3	86.00	85.06	87.33	84.67
***RCR2K1010	84.00	81.59	84.00	80.66
**ECNN2CGris3 × 3	82.33	83.92	80.00	84.67
**ECNN2CColor3 × 3	80.67	80.26	81.33	80.00

a **Image/time series processing methods used.** *Convolutional neural network obtained by neuroevolution (DeepGA). **Experimental Convolutional Neural Network. ***Time series treatment. Nomenclature of each of the treatments. *RNC2-DeepGA Convolutional neural network obtained by neuroevolution (DeepGA) 2 classes. ***GFCR2K1010150 Gaussian Frequencies with Restrictions 2 classes K = 10 window = 0.10 sigma = 150. ***GECR2K70625 Gaussian spatial with Restrictions 2 classes K = 7 window = 0.06 sigma = 2.5. **ENCE2CFourier3 × 3 Experimental Convolutional Neural Network 2 Classes gray scale applying Fourier transform 3 × 3. ***RCR2K1010 Raw with Restrictions 2 classes K = 10 windows = 0.10. **ECNN2CGris3 × 3 Experimental Convolutional Neural Network 2 Classes gray scale 3 × 3. **ECNN2CColor3 × 3 Experimental Convolutional Neural Network 2 Classes Color 3 × 3.

**Figure 10:**
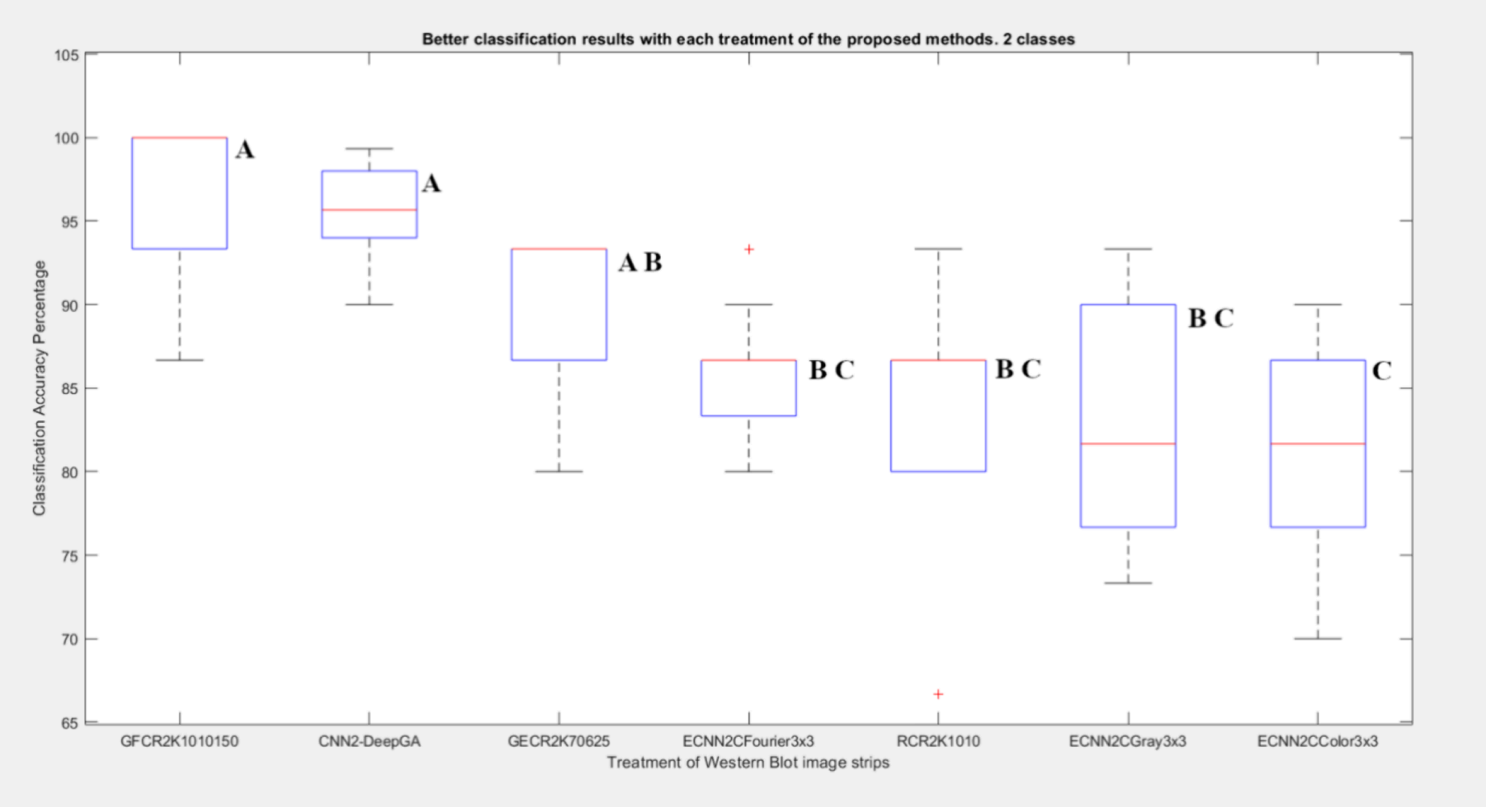
**Variability of classification of proposed methods for 2 classes.** The variability of classifications with the proposed methods with the different image treatments is shown with respect to accuracy. Box plots that do not share a letter are significantly different.

As shown in Figure 10, CNN2-DeepGA showed more consistent results due to the low variability in classification accuracy values, while ECNN2CGris3 × 3 showed a higher dispersion in classification accuracy values. ECCN2-DeepGA performed better than GFCR2K1010150 (the closest method in results) if, apart from accuracy, the results obtained in precision, sensitivity and specificity are taken into account.

## Discussion

In this work, various supervised learning and deep learning techniques were compared to analyze proteomic images, obtained from the Western Blot test of the autoantibody reaction to antigens from the T47D tumor line (ductal carcinoma). This is in order to discriminate between patients with breast cancer, with/without breast pathology.

The objective of the first experiment carried out in this work was to classify the time series obtained from Western Blot proteomic images with raw data, in an attempt to increase the classification accuracy of that obtained in Sánchez-Silva et al. (2018)^17^ and be able to compare with our results, since in the aforementioned work they use the same images as us. To achieve this increase in accuracy, it was proposed to use the Dynamic Time Warping (DTW) technique, which according to the literature,^15,30^ allows better performance in classification by obtaining the similarity between two series of dissimilar time lengths, such as those used in this work.

The best result we obtained for three classes with raw data was RCR3K506 with an accuracy of 63.56%, 63.27% precision. Without observing improvement in classification performance compared to that described in Sánchez-Silva (2018)^17^ (66.40%, 67.07%), no statistically significant difference was found (*p* < 0.05). On the other hand, for two classes RCR2K1010 was the best result with accuracy of 84.00% and 81.56% precision, being slightly below those obtained in Sánchez-Silva (2018)^17^ (86.36%, 87.31%), no statistically significant differences were found. In this experiment, the time series was used as it was obtained from the image, applying DTW as a similarity measure, unlike the geometric scaling transformation, which adds data to standardize the lengths of the time series.^27^

As a consequence of the lack of improvement in classification performance when adding the DTW as a similarity measure, it was proposed to reduce the noise of the time series using Gaussian filters, both in the space and frequency domains.^27^ The choice of the Gaussian filter is because it smoothes the image (in our case time series) without introducing changes that were not present in the original image.^31^

The first filter used was the Gaussian in the space domain, with the best result obtained for three classes with GESR3K1025 with accuracy of 63.56% and precision of 63.51%. As can be noted, the result was practically the same if compared to those obtained with the raw data (RCR3K506 63.56%, 63.27%), and lower with Sánchez-Silva (2018)^17^ (66.40%, 67.07%). No statistically significant difference was found between them as shown in Figure 9. In the experiment for two classes, with GECR2K70625 it was possible to improve the performance (90.00%, 87.63%) with respect to those obtained with raw data (RCR2K1010 84.00%, 81.56%) and those described in Sánchez-Silva (2018)^17^ (86.36%, 87.31%), there was no statistically significant difference as shown in Figure 10. Classification of the time series by applying the Gaussian filter in the space domain, as shown in previous paragraphs, failed to increase the accuracy or precision in the experiments with three classes. Meanwhile, for the experiments with two classes, accuracy and precision were improved, but they did not have a statistically significant difference.

A possible explanation for these results is the differential reaction of autoantibodies that the participants show according to the health-disease status. In the work of Romo-González et al. (2015)^18^ it was shown that patients with breast cancer have less diversity of autoantibodies, these being inherent to tumor cells, which is why the band pattern is homogeneous. This same phenomenon is observed in patients without breast pathology (healthy), who also present a minimum diversity of autoantibodies, and homogeneous and differential banding profiles from patients with breast cancer. However, autoantibodies present with greater diversity in patients with benign breast pathology, sharing certain band patterns with patients with cancer and without breast pathology.^18^ This fact could have caused the experiments with three classes to not improve the results of classification accuracy and precision. On the contrary, experiments with two classes (patients with cancer and without breast pathology) the accuracy and precision of the classification achieved good results.

In the case of the Gaussian filter in the frequency domain, the best result obtained for three classes was GFCR3K520150 with accuracy of 74.67% and precision of 75.20%. As can be seen in the results for three classes, the objective of improving the classification performance with respect to raw data (63.56%, 63.27%), those described in Sánchez-Silva et al. (2018)^17^ (66.40%, 67.07%), it was found that there was a statistically significant difference of GFCR3K520150 with respect to the other results, as can be corroborated in Figure 9. On the other hand, for two classes, the best result was obtained with GFCR2K1010150 with 96.67% accuracy, 76.35% precision, also achieving a substantial improvement in classification with respect to raw data (84.00%,81.59%), Sánchez-Silva et al. (2018)^17^ (86.36%, 87.31%) and GFCR2K1010150 was found to have a statistically significant difference from the other results, as shown in Figure 10. The improvement in classification depends largely on the type of data, some can be better classified depending on the method used for this purpose.^32^ In this experiment, the Fourier transform was used, which has been applied in the literature to find similarities between time series.^33^ In our case we have used it to transfer the time series from the space domain to the frequency domain, to apply the Gaussian smoothing filter, which improved the accuracy and precision values of the classification as can be seen in paragraphs previous.

Although the performance of time series classification was improved, especially in the frequency domain, this did not remedy the problem of selecting an area to obtain the time series and subsequently applying a filter to reduce the noise in them, which is why it is still considered a semiautomatic method. To solve this, a Convolutional Neural Network (CNN) was chosen, in which the input would be the Western Blot proteomic image.

The architecture of this CNN was designed experimentally (ECNN). The ECNN that performed best for three classes was ECNN3CGray7 × 7 with 66.89% accuracy and 67.27% precision, if these results are compared with what was obtained with the best time series classification performance (GFCR3K520150, 74.67% accuracy, 75.20% accuracy), it can be noted that the ECNN was lower in the result, but no significant differences were found as can be seen in Figure 9. For the experiments with two classes, the best result was obtained with ECNN2CFourier3 × 3 with 86.00% accuracy and 85.06% precision, if these results are compared with what was obtained with the best time series classification performance (GFCR2K1010150 with 96.67% accuracy, 76.35% precision), it can be noted that the ECNN fell below in the result, finding a statistically significant difference (Figure 10). These results obtained could be improved if the hyperparameters of the ECNN architecture are optimized to obtain better performance. To achieve the above, it was decided to use neuroevolution using the DeepGA tool,^20^ with which an ideal CNN architecture (CNN-DeepGA) was obtained for the proteomic images used in this work.

The result achieved for CNN-DeepGA for three classes was 90.67% accuracy and 90.96% precision, which if compared with the best result of the ECNN (ECNN3CGray7 × 7 with 66.89% accuracy and 67.27% precision), or series of time (GFCR3K520150 with 74.67% accuracy and 75.20% precision), it can be seen that it has the best performance of all, coupled with a significant difference with respect to the other classifications (Figure 9).

For the experiments with two classes, CNN-DeepGA obtained 95.13% accuracy and 96.65% precision which compared to the best result of ECNN (ECNN2CFourier3 × 3, 86.00% accuracy, 85.06% precision) had a better performance and with a difference significant statistic. However, compared to the best time series result (GFCR2K1010150, 96.67% accuracy, 76.35% precision) it can be noted that the performance of CNN-DeepGA was lower, but without a significant statistical difference (Figure 10).

The use of ECNN allowed to explore the classification of Western Blot proteomic images automatically, although the results were not entirely successful. However, this was reversed due to the obtaining of an optimized architecture using the DeepGA algorithm, thus achieving automation and high performance in image classification. In this sense, the evaluation of the techniques that come from supervised learning, which is a subarea of machine learning, proposed in this study, allowed not only the discrimination between patients with breast pathology (cancer), benign breast pathology and without pathology (healthy), through the analysis and classification of Western Blot proteomic images for the early detection of breast cancer, but also to compare the performance of the chosen classifiers (the K-nearest neighbors algorithm and convolutional neural networks obtained experimentally and by neuroevolution). With this, we can confirm that to classify proteomic images (Western Blot) for the discrimination between patients for the timely detection of breast cancer, it was found that the best performance was obtained with a convolutional neural network obtained by neuroevolution.

Like all computer algorithms in the medical field, these supervised learning techniques that have been evaluated serve as a reference for decision-making, since the person who has to make the decision when issuing a medical diagnosis is the expert in that area.

This work gives us an approach to the analysis of proteomic images with various supervised learning techniques. However, it is necessary to carry out more experiments with a larger number of images of serums from patients whose diagnosis is unknown and to monitor them to evaluate their predictive power. That is, these new images would be analyzed with CNN-Deep-GA and these patients would be monitored to verify the theory that breast cancer can be detected early with the reaction of the immune system to tumor antigens. Therefore, for us to have a tool available in a medical institution, it will take some time.

This work gives us an approach to the analysis of proteomic images with several supervised learning techniques. However, it is necessary to carry out more experiments with a larger number of images of serums from patients whose diagnosis is unknown and monitor them to evaluate their predictive power. That is, these new images would be analyzed with CNN-Deep-GA and these patients would be monitored and the theory that breast cancer can be detected early with the reaction of the immune system to tumor antigens would be tested. On the other hand, to add interpretability to the prediction made by CNN-Deep-GA, it is suggested to use the Grad-CAM technique^34^ as future work, which would allow us to know which parts of the strips are significant to discriminate the classes of patients. This could generate confidence in public opinion about the suggestions that this type of algorithms make for decision making. With all this, it will take some time for us to have a tool available in some medical institution.

## References

[ref1] World Health Organization. Data visualization tools for exploring the global cancer burden in 2022. Cancer Today. https://gco.iarc.fr/today/en/dataviz/pie?mode=cancer.

[ref2] Palmero Picazo J.; Lassard Rosenthal J.; Juárez Aguilar L. A.; Medina Núñez C. A. Cáncer de mama: una visión general. Acta Médica Grupo Ángeles 2021, 19 (3), 354–360. 10.35366/101727.

[ref3] Collado R. HABLEMOS DE El Cáncer de Mama Con Roche; Hablemos de; ACV, Activos de Comunicación Visual, S.A., 2011.

[ref4] Amens J. N.; Bahçecioglu G.; Zorlutuna P. Immune System Effects on Breast Cancer. Cell. Mol. Bioeng. 2021, 14 (4), 279–292. 10.1007/s12195-021-00679-8. 34295441 PMC8280260

[ref5] Ekici S.; Jawzal H. Breast Cancer Diagnosis Using Thermography and Convolutional Neural Networks. Med. Hypotheses 2020, 137, 109542 10.1016/j.mehy.2019.109542. 31901878

[ref6] Cárdenas-Sánchez J. Consenso mexicano sobre diagnóstico y tratamiento del cáncer mamario. Gac. Mex. Oncol. 2022, 20 (92), 6923 10.24875/j.gamo.M21000213.

[ref7] Qiu J.; Keyser B.; Lin Z.-T.; Wu T. Autoantibodies as Potential Biomarkers in Breast Cancer. Biosensors 2018, 17, 67 10.3390/bios8030067. PMC6163859 30011807

[ref8] Rauf F.; Anderson K. S.; LaBaer J. Autoantibodies in Early Detection of Breast Cancer. Cancer Epidemiol. Biomarkers Prev. 2020, 29 (12), 2475–2485. 10.1158/1055-9965.EPI-20-0331. 32994341 PMC7710604

[ref9] Wang L. Early Diagnosis of Breast Cancer. Sensors 2017, 17 (7), 1572 10.3390/s17071572. 28678153 PMC5539491

[ref10] Yang R.; Han Y.; Yi W.; Long Q. Autoantibodies as Biomarkers for Breast Cancer Diagnosis and Prognosis. Front. Immunol. 2022, 13, 1035402 10.3389/fimmu.2022.1035402. 36451832 PMC9701846

[ref11] Yadav S.; Kashaninejad N.; Masud M. K.; Yamauchi Y.; Nguyen N.-T.; Shiddiky M. J. A. Autoantibodies as Diagnostic and Prognostic Cancer Biomarker: Detection Techniques and Approaches. Biosens. Bioelectron. 2019, 139, 111315 10.1016/j.bios.2019.111315. 31132724

[ref12] Chougrad H.; Zouaki H.; Alheyane O. Deep Convolutional Neural Networks for Breast Cancer Screening. Comput. Methods Programs Biomed. 2018, 157, 19–30. 10.1016/j.cmpb.2018.01.011. 29477427

[ref13] Zhang M.; Sadinski M.; Haddad D.; Bae M. S.; Martinez D.; Morris E. A.; Gibbs P.; Sutton E. J. Background Parenchymal Enhancement on Breast MRI as a Prognostic Surrogate: Correlation With Breast Cancer Oncotype Dx Score. Front. Oncol. 2021, 10, 595820 10.3389/fonc.2020.595820. 33614481 PMC7890019

[ref14] Zhu H.; Zhao P.; Chan Y.-P.; Kang H.; Lee D. L. Breast Cancer Early Detection with Time Series Classification. In Proceedings of the 31st ACM International Conference on Information & Knowledge Management; ACM: Atlanta GA USA, 2022; pp 3735–3745. 10.1145/3511808.3557107.

[ref15] Gardezi S. J. S.; Faye I.; Sanchez Bornot J. M.; Kamel N.; Hussain M. Mammogram Classification Using Dynamic Time Warping. Multimed. Tools Appl. 2018, 77 (3), 3941–3962. 10.1007/s11042-016-4328-8.

[ref16] Abunasser B.; AL-Hiealy M. R.; Zaqout I.; Abu-Naser S. Convolution Neural Network for Breast Cancer Detection and Classification Using Deep Learning. Asian Pac. J. Cancer Prev. 2023, 24 (2), 531–544. 10.31557/APJCP.2023.24.2.531. 36853302 PMC10162639

[ref17] Sánchez-Silva D. M.; Acosta-Mesa H. G.; Romo-González T. Semi-Automatic Analysis for Unidimensional Immunoblot Images to Discriminate Breast Cancer Cases Using Time Series Data Mining. Int. J. Pattern Recognit. Artif. Intell. 2018, 32 (01), 1860004 10.1142/S0218001418600042.

[ref18] Romo-González T.; Esquivel-Velázquez M.; Ostoa-Saloma P.; Lara C.; Zentella A.; León-Díaz R.; Lamoyi E.; Larralde C. The Network of Antigen-Antibody Reactions in Adult Women with Breast Cancer or Benign Breast Pathology or without Breast Pathology. PLoS One 2015, 10 (3), e0119014 10.1371/journal.pone.0119014. 25781932 PMC4363365

[ref19] Llaguno-Roque J.-L.; Barrientos-Martínez R.-E.; Acosta-Mesa H.-G.; Romo T. Western Blot Pattern Classification Using Convolutional Neural Networks for Breast Cancer Diagnosis. Proc. 4th Workshop New Trends Comput. Intell. Appl. CIAPP 2022 2022.

[ref20] Vargas-Hákim G.-A.; Mezura-Montes E.; Acosta-Mesa H.-G. Hybrid Encodings for Neuroevolution of Convolutional Neural Networks: A Case Study; Association for Computing MachineryNew YorkNYUnited States: Lille, France, 2021; pp 1762–1770. 10.1145/3449726.3463133.

[ref21] Llaguno-Roque J.-L.; Barrientos-Martínez R.-E.; Acosta-Mesa H.-G.; Romo-González T.; Mezura-Montes E. Neuroevolution of Convolutional Neural Networks for Breast Cancer Diagnosis Using Western Blot Strips. Math. Comput. Appl. 2023, 28 (3), 72 10.3390/mca28030072.

[ref22] Pérez-Hernández J.; León-Díaz R.; Zentella A.; Lamoyi E.; Esquivel-Velázquez M.; Barranca-Enríquez A.; Romo-González T. Autoantibody Diversity Is Augmented in Women with Breast Cancer and Is Related to the Stage of the Disease. Curr. Oncol. 2023, 30 (10), 8793–8804. 10.3390/curroncol30100634. 37887534 PMC10605201

[ref23] Tan P.-N.; Steinbach M.; Kumar V. Introduction to Data Mining; Pearson, 2005.

[ref24] Sakoe H.; Chiba S. Dynamic Programming Algorithm Optimization for Spoken Word Recognition. IEEE 1978, 26 (1), 43–49. 10.1109/TASSP.1978.1163055.

[ref25] Cassisi C.; Montalto P.; Aliotta M.; Cannata A.; Pulvirenti A. Similarity Measures and Dimensionality Reduction Techniques for Time Series Data Mining. In Advances in Data Mining Knowledge Discovery and Applications; Karahoca A., Ed.; InTech, 2012. 10.5772/49941.

[ref26] Keogh E.; Lonardi S.; Ratanamahatana C. A. Towards Parameter-Free Data Mining. In Proceedings of the 2004 ACM SIGKDD international conference on Knowledge discovery and data mining - KDD’04; ACM Press: Seattle, WA, USA, 2004; p 206. 10.1145/1014052.1014077.

[ref27] González R. C.; Woods R. E.; Masters B. R. Digital Image Processing. Third Edition. J. Biomed. Opt. 2009, 14 (2), 029901 10.1117/1.3115362.

[ref28] Sarvamangala D. R.; Kulkarni R. V. Convolutional Neural Networks in Medical Image Understanding: A Survey. Evol. Intell. 2022, 15 (1), 1–22. 10.1007/s12065-020-00540-3. 33425040 PMC7778711

[ref29] Goodfellow I.; Bengio Y.; Courville A. Deep Learning; MIT Publisher, 2016.

[ref30] Kate R. J. Using Dynamic Time Warping Distances as Features for Improved Time Series Classification. Data Min. Knowl. Discovery 2016, 30 (2), 283–312. 10.1007/s10618-015-0418-x.

[ref31] Marr D.; Ullman S. Vision: A Computational Investigation into the Human Representation and Processing of Visual Information; MIT Press: Cambridge, Mass., 2010.

[ref32] Ding H.; Trajcevski G.; Scheuermann P.; Wang X.; Keogh E. Querying and Mining of Time Series Data: Experimental Comparison of Representations and Distance Measures. Proc. VLDB Endow. 2008, 1 (2), 1542–1552. 10.14778/1454159.1454226.

[ref33] Bagnall A.; Lines J.; Bostrom A.; Large J.; Keogh E. The Great Time Series Classification Bake off: A Review and Experimental Evaluation of Recent Algorithmic Advances. Data Min. Knowl. Discovery 2017, 31 (3), 606–660. 10.1007/s10618-016-0483-9. PMC6404674 30930678

[ref34] Selvaraju R. R.; Cogswell M.; Das A.; Vedantam R.; Parikh D.; Batra D. Grad-CAM: Visual Explanations from Deep Networks via Gradient-Based Localization. Int. J. Comput. Vis. 2020, 128 (2), 336–359. 10.1007/s11263-019-01228-7.

